# Sustained positive consequences of genetic rescue of fitness and behavioural traits in inbred populations of *Drosophila melanogaster*

**DOI:** 10.1111/jeb.14015

**Published:** 2022-05-09

**Authors:** Daniel Bang Jørgensen, Michael Ørsted, Torsten Nygaard Kristensen

**Affiliations:** ^1^ 1004 Department of Chemistry and Bioscience Aalborg University Aalborg E Denmark; ^2^ Department of Biology Aarhus University Aarhus C Denmark

**Keywords:** behaviour, biodiversity, donor populations, *Drosophila melanogaster*, fitness, genetic rescue, genetic variation, inbreeding depression, negative geotaxis, population fragmentation

## Abstract

One solution to alleviate the detrimental genetic effects associated with reductions in population size and fragmentation is to introduce immigrants from other populations. While the effects of this genetic rescue on fitness traits are fairly well known, it is less clear to what extent inbreeding depression and subsequent genetic rescue affect behavioural traits. In this study, replicated crosses between inbred lines of *Drosophila melanogaster* were performed in order to investigate the effects of inbreeding and genetic rescue on egg‐to‐adult viability and negative geotaxis behaviour—a locomotor response used to measure, e.g. the effects of physiological ageing. Transgenerational effects of outcrossing were investigated by examining the fitness consequences in both the F_1_ and F_4_ generation. The majority of inbred lines showed evidence for inbreeding depression for both egg‐to‐adult viability and behavioural performance (95% and 66% of lines, respectively), with inbreeding depression being more pronounced for viability compared with the locomotor response. Subsequent outcrossing with immigrants led to an alleviation of the negative effects for both viability and geotaxis response resulting in inbred lines being similar to the outbred controls, with beneficial effects persisting from F_1_ to F_4_. Overall, the results clearly show that genetic rescue can provide transgenerational rescue of small, inbred populations by rapidly improving population fitness components. Thus, we show that even the negative effects of inbreeding on behaviour, similar to that of neurodegeneration associated with physiological ageing, can be reversed by genetic rescue.

## Graphical abstract

From 150 inbred lines of Drosophila melanogaster, donor and recipient populations was selected to perform genetic rescue crosses. The negative consequences of inbreeding depression was alleviated for both egg‐to‐adult viability and a behavioural locomotor trait in the F_1_ and F_4_ generations.

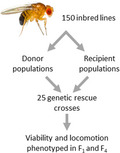

## 1 INTRODUCTION

To manage the detrimental genetic effects associated with population fragmentation, such as inbreeding and loss of genetic variation (Bijlsma & Loeschcke, 2012; Bouzat, 2010; Frankham et al., 2002; Hedrick & Fredrickson, 2010; Hedrick & Kalinowski, 2000; López‐Cortegano et al., 2019; Ørsted et al., 2019, 2022; Reed, 2004), introducing immigrants from other populations, termed ‘genetic rescue’, is increasingly being considered as a management approach (Hoffmann et al., 2021a, 2021b; Ingvarsson, 2001; Tallmon et al., 2004; Weeks et al., 2011; Whiteley et al., 2015; Willi et al., 2022). Several examples have proven that genetic rescue can restore/increase fitness and reduce the extinction risk of small genetically depauperate natural populations (Bouzat et al., 2009; Hedrick & Fredrickson, 2010; Hoffmann et al., 2021a; Hogg et al., 2006; Madsen et al., 1999; Weeks et al., 2017; Westemeier, 1998). Similarly, numerous experimental studies support the promising potential of genetic rescue in laboratory populations (Ball et al., 2000; Bijlsma et al., 2010; Bryant et al., 1999; Heber et al., 2012; Holleley et al., 2011; Hufbauer et al., 2015; Jensen et al., 2018; Spielman & Frankham, 1992; Swindell & Bouzat, 2006; Waite et al., 2005).

To increase the probability of successful implementation of genetic rescue, guidelines on the appropriate procedures for genetic rescue planning and management have been developed (Frankham et al., 2011; Hedrick & Fredrickson, 2010; Hoffmann et al., 2021a; Weeks et al., 2011). For the continuous improvement of these guidelines, lessons learned from experimental studies can be useful. However, most laboratory studies have examined the impacts of inbreeding and genetic rescue on fitness traits, such as survival or reproduction (Bijlsma et al., 2010; Bryant et al., 1999; Holleley et al., 2011; Hufbauer et al., 2015; Jensen et al., 2018; Joubert & Bijlsma, 2010; Kristensen et al., 2008, 2011; Mikkelsen et al., 2010; Schou et al., 2015; Spielman & Frankham, 1992; Waite et al., 2005), and we are unaware of studies examining the effects of genetic rescue on behavioural traits. Nonetheless, knowledge of this may provide crucial insight into the effects of population fragmentation and immigration, since the fitness consequences of outcrossing are not universal for all components of fitness and because behaviour affects species persistence through a wide variety of mechanisms (Reed, 1999; Whiteley et al., 2015).

Although studies have provided evidence for the beneficial effects of genetic rescue, studies investigating the effects beyond the first generation (F_1_) are rare (for a review see Edmands, 2007). Knowledge of the long‐term impact of genetic rescue is important since the effect of gene flow on fitness may vary across generations. Heterosis is expected to peak in the F_1_ generation (due to maximum heterozygosity), followed by a decline in the later generations due to the re‐accumulation of genetic load and possible expression of outbreeding depression (Edmands, 2007; Tallmon et al., 2004; Waller, 2015; Whiteley et al., 2015). Consequently, whether the introduction of immigrants leads to genetic restoration may depend on the relative importance of heterosis and outbreeding depression (Bell et al., 2019; Tallmon et al., 2004; Whiteley et al., 2015) and may not persist across generations.

In this study, we examined the effect of genetic rescue on egg‐to‐adult viability and negative geotaxis, with the latter being a behavioural response that has frequently been used to assess age‐related declines in locomotor activity (Grotewiel et al., 2005). A total of 150 lines of *Drosophila melanogaster* were subjected to three generations of full‐sib mating, whereafter the two traits were assessed. Next, to represent a hypothetical genetic rescue scenario, five populations with low fitness (recipient populations) were selected to be rescued by immigration from five populations with high fitness (donor populations). For each of the recipient populations, the genetic rescue was carried out by introducing immigrants from each of the donor populations individually, followed by an assessment of the fitness components in the F_1_ and F_4_ generation. The study aimed at testing three hypotheses: (1) Inbreeding through consecutive full‐sib mating impacts negatively on both fitness components investigated, i.e. inbreeding depression is evident for both egg‐to‐adult viability and the behavioural trait. (2) Inbreeding depression is more pronounced for egg‐to‐adult viability compared with negative geotaxis response. (3) Outcrossing with immigrants leads to heterosis in both traits, which peaks in the F_1_ generation followed by a decline in later generations.

## 2 MATERIALS AND METHODS

### 2.1 Fly stock and maintenance

The mass population of *D*. *melanogaster* used in this study was established by crossing five mass‐bred populations collected at Karensminde orchard, Denmark (55°56042.46″N, 10°12045.31″E), in the time period 2010–2017. The original mass‐bred populations were established from, respectively, 589, 20, 25, 20 and 25 inseminated females. They were held under standard laboratory conditions; 20°C, 50% RH, 12:12 light:dark photoperiod, and placed on the standard *Drosophila* medium (water 1 L/L, sugar 40 g/L, oatmeal 30 g/L, agar 16 g/L and dry yeast 60 g/L, with the addition of nipagen (12 ml/L) and 80% acetic acid (1.2 ml/L) to control for fungal growth) at minimum 1000 individuals per generation. In the fall of 2020, approximately 400 flies from each mass‐bred population were mixed to establish a new mass‐bred population. This was done in order to increase genetic variation in the experimental population. The new mass‐bred population was maintained at a minimum size of 1000 individuals distributed in five 236‐mL bottles with a 75‐mL medium. The newly established mass population was kept at 23 ± 1°C, 50% RH and a 12:12 photoperiod for five generations prior to initiating the inbreeding procedure.

### 2.2 Inbreeding procedure

**FIGURE 1:**
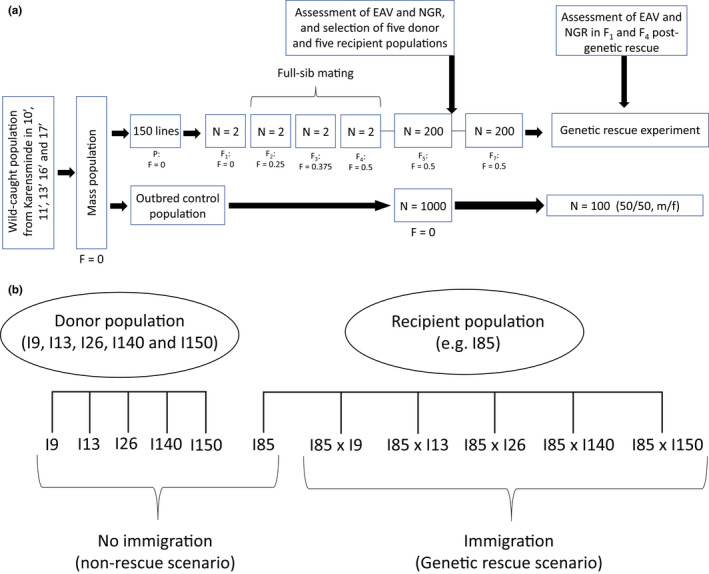
Flowchart of the experimental procedure. (a) The experimental procedure beginning with the establishment of the mass population and the inbreeding procedure on 150 lines (three generations of full‐sib mating), followed by the assessment of egg‐to‐adult viability (EAV) and negative geotaxis response (NGR), and subsequent selection of the five donor and five recipient populations to be used in the genetic rescue experiment. (b) Hereafter, the genetic rescue experiment was initiated. Each recipient population (here illustrated for population I85) was “rescued” by immigration from each individual donor population (I9, I13, I26, I140 or I150). EAV and NGR were assessed again in the F_1_ and F_4_ generation, after initiation of the genetic rescue experiment, to assess the effect of rescue on fitness. The rescue effect was assessed by calculating the mid‐parent heterosis (MPH), describing the percentual superiority of the hybrid offspring compared with the mid‐parent fitness. See text for details on each step in the procedure

A total of 150 inbred lines were established from the mass‐bred population by three consecutive generations of full‐sib mating, reaching an expected inbreeding level of *F* = 0.5 (Figure 1a; Falconer & Mackay, 1996). To establish each inbred line and subsequent full‐sib generations, one virgin male and one virgin female were randomly collected less than eight hours after emergence and placed together in a single vial for 48 h to allow mating. Simultaneously with initiating the inbreeding treatments, five replicate outbred control lines (*F* = 0) were established by increasing the mass population to a population size of minimum of 5000 individuals and distributing 1000 individuals to each control line. Each control line was maintained in five 236‐ml bottles. Due to the loss of individuals or unsuccessful reproduction, some lines were lost during the inbreeding procedure, leaving 110 inbred lines and five outbred control lines to be phenotyped and used in the genetic rescue experiment. To minimize further inbreeding in the 110 inbred lines, each line was increased to approximately 200 individuals.

### 2.3 Genetic rescue experiment

To study the effect of immigration on the fitness of an inbred population, five inbred lines (I9, I13, I26, I140 and I150; denoted “donor populations”) were chosen as immigrants that were used to “rescue” five different inbred lines (I85, I117, I132, I143 and I148; denoted “recipient populations”). The five recipient and five donor populations used in the genetic rescue crossing experiment were chosen from the 110 inbred lines based on their median and variation in egg‐to‐adult viability visualized in a boxplot (Figure S1). The five lines with the lowest median and smallest range in viability were selected as recipient populations, and the five lines with the highest median and smallest range in viability were selected as donor populations.

Five genetic rescue crosses were set up for each of the five recipient populations, for a total of 25 crosses (Figure 1b). 50 virgin adult males from each of the five donor populations were individually crossed with 50 virgin females from each of the five recipient populations to assess the genetic rescue effect of each individual donor population. Simultaneously, each donor, recipient and control line was maintained uncrossed at a density of 100 individuals (50 males and 50 females) and tested in the same generations as the crossed lines. Flies from all crosses were maintained in 170‐ml bottles with a 50‐ml medium. Males and females were kept in separate bottles for a maximum of 72 h prior to setting up the crosses, and both males and females were 48 ± 24 h old when allowed to mate.

### 2.4 Phenotypes assessed

Two fitness components were assessed: Egg‐to‐adult viability (EAV) and negative geotaxis (hereafter referred to as ‘negative geotaxis response’; NGR).

### 2.5 Egg‐to‐adult viability (EAV)

Egg‐to‐adult viability was assessed in the 110 inbred lines and five control lines by allowing approximately 100 3–6 days old flies to lay eggs in a 236‐ml bottle with a 25‐ml coloured medium to facilitate the counting of eggs. After 12 h, 20 eggs were collected and placed in each of five vials with a 7.5‐ml medium. EAV for all F_1_ and F_4_ crosses used in the genetic rescue crossing experiment and the five control lines were assayed in a similar manner; 100 3–6 days old flies were allowed to lay eggs, whereafter 20 eggs were collected and placed in each of 10 vials per cross in both F_1_ and F_4_. The number of emerged adults was recorded daily until no flies emerged for two consecutive days. EAV was estimated as the proportion of individuals that successfully enclosed.

### 2.6 Negative geotaxis response (NGR)

To assess the impact of inbreeding and genetic rescue on a behavioural trait related to locomotion, male flies that enclosed in the EAV assay were also assayed for NGR. Thus, flies tested for NGR had developed at a controlled density of no more than 20 individuals per vial. The NGR was assessed using a modified version of the Rapid Iterative Negative Geotaxis (RING) assay, originally developed by Gargano et al. (2005) to study age‐related declines in locomotor activity of flies. By utilizing mechanical stimulation to tap a replicate number of flies to the bottom of an empty vial, the NGR was stimulated and the flies began to ascend the sides of the vial. The ascending distance moved by the flies was then recorded using digital photography, and flies ascending a larger distance were interpreted as being more active, which in turn was assumed to be a fitness benefit. To record the response of multiple groups of flies simultaneously, the RING assay was carried out in a RING apparatus (for details on the RING apparatus used for this assay, see Figure S2).

NGR was assessed using 1–4 days old male flies from 106 of the 110 surviving inbred lines (four inbred lines assessed for EAV failed to generate enough males to perform the RING assay) and from the five control lines by transferring a total of five male flies into each of three empty vials per line. For all genetic rescue crosses and the five control lines, six vials were assayed in both F_1_ and F_4_. After the flies were transferred to the vials, each vial was inserted into the RING apparatus (holding ten vials). The flies were then allowed to adjust to the new empty vials for one minute before beginning the RING assay. The RING apparatus was subsequently knocked down forcefully on a table in three rapid successions to initiate the NGR, and a photograph of the vertical position of each fly in the RING apparatus was captured exactly three seconds after the 3rd knockdown of the RING apparatus. A camera‐timer of 3 s was used as Ørsted et al. (2017) found the three‐second time frame to provide the most informative data. The RING assay was run a total of five times per group (five trials) with 30 s intermissions, resulting in a total of five images of each vial. The position of the flies in the RING apparatus was captured 30 cm from the apparatus using an iPhone 11 (12 Mp; Apple Inc.) and was analyzed manually using ImageJ software (version 1.8.0_172; Rasband, 2020). The vertical distance moved by the flies, ascending from the base of the vial, was noted in centimetres (cm). Since the height of the vial was 6 cm, NGR values had a range from 0 to 6 (cm). All images of the flies were scaled in height according to an invariant landmark in order to standardize all measurements of the position of the flies. The assay was run in a climate‐controlled room at 23 ± 1°C between 08:00 and 12:00 on each test day to minimize the impact of the circadian rhythm of the flies on locomotor activity (Allada et al., 2001).

### 2.7 Inbreeding depression and genetic rescue effect

To assess inbreeding depression in the investigated traits, all 110 inbred lines and the five outbred control lines were assayed before initiating the genetic rescue crosses. Inbreeding depression (δ) in EAV and NGR was estimated as the proportional reduction in the mean value of an individual inbred line (i) compared with the mean of the five outbred control lines (Fox & Reed, 2011):

$$\delta_{i}=\frac{{\text{mean}}_{\text{control}}‐{\text{mean}}_{\text{inbred},i}}{{\text{mean}}_{\text{control}}}$$

To allow comparisons of the effects of inbreeding among studies, traits and taxa, we computed the inbreeding depression rate (B) for the 110 inbred lines. This was calculated as the number of haploid lethal equivalents (Hedrick, 2011):

$$B=‐\frac{1}{f}\ln\left(\frac{w_{f}}{w_{o}}\right)$$

where f is the inbreeding coefficient (set to *f* = 0.5), and w_f_ and w_o_ are the mean fitness of inbred and outbred (control) individuals, respectively. B describes the rate at which fitness changes with increased inbreeding, with B > 0 denoting a decline in fitness as the inbreeding level increases. When there is no inbreeding depression, B is equal to 0. By accounting for the inbreeding coefficient, the inbreeding depression rate provides a measure of inbreeding depression, whereby the effect of different levels of inbreeding is standardized.

To assess the effect of genetic rescue, all donor and recipient populations, all established crosses between donor and recipient populations and all five control lines were assessed using the same assays (EAV and NGR). This was carried out in the F_1_ and F_4_ generation after the genetic rescue crosses were set up. The benefit of genetic rescue (i.e. heterosis) was calculated as the mid‐parent heterosis (MPH), which describes the percentual superiority of the hybrid offspring compared with the mid‐parent fitness (MP). Mid‐parent heterosis was calculated according to Solieman et al. (2013) as:

$$\text{MPH}=\frac{\left(F_{i}‐\text{MP}\right)}{\text{MP}}*100$$

where F_i_ is the fitness value of hybrid (i.e. rescued) individuals in the i'th generation, produced by crossing recipient and donor populations, and MP is the mean fitness value of the two parental populations in each genetic rescue cross (one recipient and one donor population).

### 2.8 Statistical analysis

The NGR of the flies within each vial was calculated as the median distance ascended by the five flies per trial, generating one data point for each of the five trials. The average response across all trials was then calculated as the average of these five data points. A small number of lines (<8%) showed a small but significant effect on trial number, i.e. a reduced NGR with the increasing number of trials, therefore a correction in NGR was made for these lines (see Table S1 for details).

To investigate recipient population and generation effects on genetic rescue, we fitted generalized linear mixed effect models (GLMMs) in the R‐package ‘lme4’ (Bates et al., 2015). For EAV we assumed a binomial distribution with a logit link function, while for NGR, a regular Poisson distribution was assumed. We fitted either trait as the response variable as a function of generation (Pre‐GR, F_1_ and F_4_), and recipient population and their interaction as fixed effects, while donor population was included as a random effect. These full models were compared with individual models without the recipient population fixed effect by *χ* ^2^ difference tests. Conditional coefficients of determination of the GLMMs interpreted as the variance explained by the entire model, including both fixed and random effects, were calculated as $R_{\text{GLMM}\left(c\right)}^{2}=\frac{\sigma_{f}^{2}+\sigma_{\alpha }^{2}}{\sigma_{f}^{2}+\sigma_{\alpha }^{2}+\sigma_{\varepsilon }^{2}}$, where $\sigma_{f}^{2},\sigma_{\alpha }^{2},\sigma_{\varepsilon }^{2}$ are the variances of the fixed effect components, the random effects and the residual variance, respectively (see ‘delta‐method’ in Nakagawa et al., 2017). To test whether mid‐parent heterosis was significant, i.e. whether hybrid offspring fitness was significantly greater than mid‐parent fitness, we used a one‐tailed one‐sample Wilcoxon signed‐rank test. To test whether mid‐parent heterosis was significantly different in F_1_ and F_4_, we used a two‐sample paired Wilcoxon signed‐rank test. Correlations between EAV and NGR were estimated by calculating the Spearman rank correlation coefficient. All statistical analyses were performed in R (v. 3.5.1; R Core Team, 2020).

## 3 RESULTS

### 3.1 Inbreeding depression for fitness and locomotion

The majority of the 110 inbred lines showed evidence of inbreeding depression in both EAV and NGR prior to the genetic rescue experiment. Of the inbred lines, 104 (95%) showed inbreeding depression in EAV (δ > 0), while 70 of the inbred lines (66%) showed inbreeding depression in NGR (Figure S3; Jørgensen et al., 2022). Averaged across all lines, the inbred lines showed a ~26% reduction in EAV (mean ± SE: Control = 0.882 ± 0.017; Inbred = 0.652 ± 0.010) and a ~10% reduction in NGR (mean ± SE: Control = 3.860 ± 0.115; Inbred = 3.464 ± 0.056), compared with outbred control lines. This reduction was significant for both fitness components (EAV: *t*_41.635_ = 11.744, *p* < 0.001; NGR: *t*_17.905_ = 2.404, *p* = 0.027). Across all inbred lines, the mean inbreeding depression rate (B) was higher for EAV compared with NGR (mean ± SE: EAV = 0.742 ± 0.083; NGR = 0.282 ± 0.048).

To assess the potential relationship between inbreeding depression in EAV and NGR, we calculated the correlation between measures of inbreeding depression for the two traits. Across all generations, inbreeding depression in EAV was positively correlated with inbreeding depression (*r*_s_ = 0.337, *p* < 0.001), which was also the case when estimating the correlation between EAV and NGR within each generation (Pre‐GR, F_1_ and F_4_) (Table S2). The amount of inbreeding depression observed in the two fitness traits was significantly different across all investigated generations, with the median inbreeding depression across all inbred lines being higher for EAV, compared with NGR, in all cases (Table S3).

### 3.2 Positive and sustained effects of genetic rescue

**FIGURE 2:**
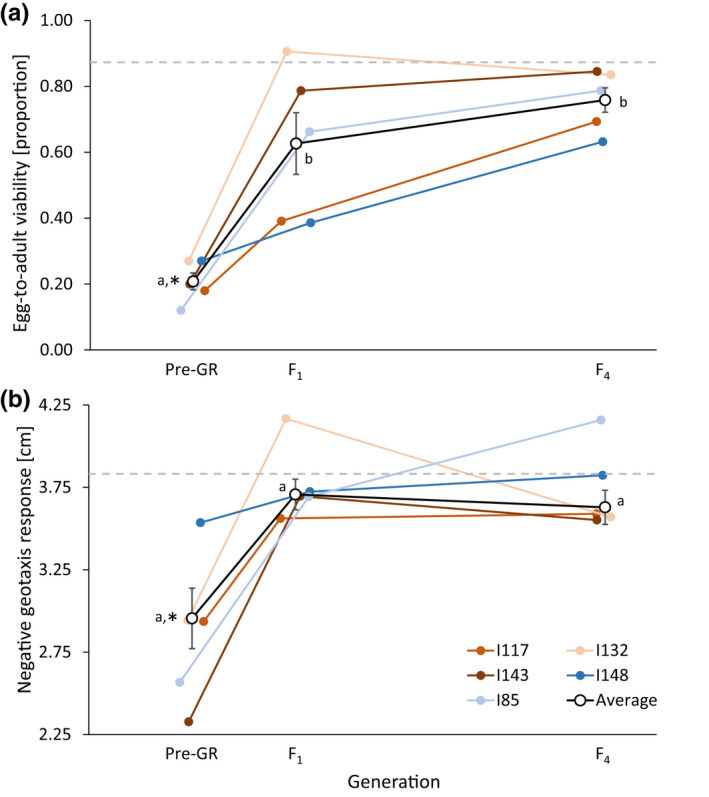
Transgenerational effect of genetic rescue on mean fitness for the two investigated traits; (a) egg‐to‐adult viability (EAV) and (b) negative geotaxis response (NGR). Values are averaged across all recipient populations in the generation prior to genetic rescue (Pre‐GR) and averaged across all genetic rescue crosses in the F_1_ and F_4_ generation. Open circles and the black lines are average across all recipient populations (error bars are SE; *n* = 5), while colours represent individual recipient populations (same in both panels, error bars omitted). Random jitter has been introduced on the x‐axis for increased visibility. Letters denote significant differences between generations based on pairwise comparisons using the Wilcoxon rank‐sum test (*p* < 0.05), while an asterisk denotes that the average across recipient populations are significantly different from the average of the five control lines (dashed grey lines; one‐tailed one‐sample Wilcoxon signed‐rank test) tested in all three generations; Pre‐GR, F_1_ and F_4_, i.e. for both traits, the average across the five recipient populations were similar to that of the outbred control lines

Averaged across all recipient populations, genetic rescue resulted in a significant increase in fitness in the F_1_ generation for both traits, with a 201% and 24% increase in EAV and NGR, respectively, compared with fitness values prior to genetic rescue. These beneficial effects were sustained throughout the F_4_ generation, as there was no significant difference in either trait between F_1_ and F_4_ (Figure 2 and Table S5). For both traits, average values across the five recipient populations in both F_1_ and F_4_ were not significantly different from that of the outbred control lines (Figure 2). These effects were consolidated by the results of the generalized linear mixed models showing strong effects of generation and recipient population and a significant interaction for EAV (Table 1). For NGR, there was no significant effect of generation, but a strong effect on the recipient population and a significant interaction between generation and recipient population, suggesting population‐specific transgenerational effects. For both traits including the recipient population as an explanatory variable improved the fit of the models (Table 1).

**TABLE 1:** Results of the general linear mixed models (GLMMs) of egg‐to‐adult viability (EAV; top) and negative geotaxis response (NGR; bottom) as a function of generation (Pre‐GR, F_1_ and F_4_) and recipient population (rec_pop) and their interaction as fixed effects, and donor populations as a random effect

Trait	Fixed effects	*χ* ^2^	*df*	*p*
EAV	(Intercept)	15.44	1	<.001***
	Generation	156.29	2	<.001***
	rec_pop	1032.87	9	<.001***
	Generation*rec_pop	220.74	18	<.001***

	Random effects	*SD*		
	Donor population	1.07		

	Comparison with model without rec_pop	*χ* ^2^ *df*	*χ* ^2^	*p*
		1473.21	27	<0.001***

Trait	Fixed effects	χ^2^	*df*	*p*
NGR	(Intercept)	452.00	1	<0.001***
	Generation	0.87	2	0.646
	rec_pop	23.59	9	0.005**
	Generation*rec_pop	35.59	18	0.008**

	Random effects	*SD*		
	Donor population	0.35		

	Comparison with model without rec_pop	χ^2^ *df*	*χ* ^2^	*p*
		49.60	27	0.005**

These full models were compared with individual models without the recipient population fixed effect by χ^2^ tests. Conditional coefficients for the determination of the GLMMs $\left(R_{\mathrm{full}\;\mathrm{model}}^{2}\right)$ interpreted as the variance explained by the entire model, including both fixed and random effects, were 0.33 and 0.35 for EAV and NGR, respectively. Asterisks denote the significance of individual variables or interactions; ***p < 0.001; **p < 0.01; and *p < 0.05.

**FIGURE 3:**
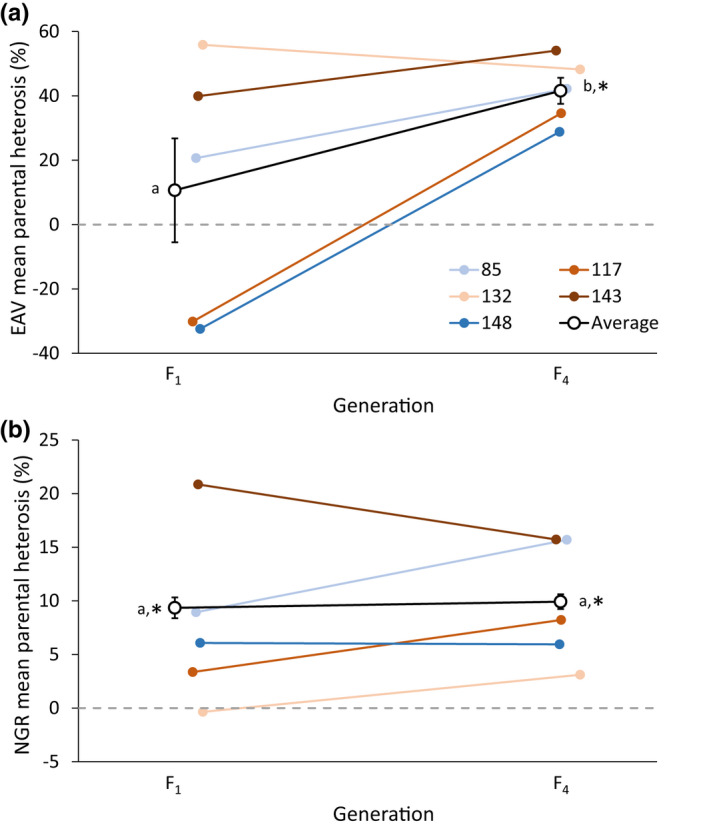
Transgenerational effects of genetic rescue on mean mid‐parent heterosis (MPH) for the two traits; (a) egg‐to‐adult viability (EAV) and (b) negative geotaxis response (NGR), note different scales on y‐axis. Values are averaged across all recipient populations in the two generations following genetic rescue (F_1_ and F_4_). Open circles and the black lines are average across all recipient populations (error bars are SE; *n* = 5), while colours represent individual recipient populations (same in both panels, error bars omitted). Random jitter has been introduced on the x‐axis for increased visibility. Values are presented in Table S6. Letters denote significant differences between generations based on pairwise comparisons using the Wilcoxon rank‐sum test (*p* < 0.05), while an asterisk denotes that the average across recipient populations are significantly different from 0 (dashed grey lines; one‐tailed one‐sample Wilcoxon signed‐rank test), i.e. testing for offspring fitness being greater than mid‐parent fitness

Genetic rescue showed significant beneficial effects for EAV, as the number of crosses showing significant MPH (i.e. hybrid offspring fitness higher than mid‐parent fitness) in F_1_ was 13 (52%), while 21 (84%) crosses showed significant MPH in F_4_. Similarly, beneficial effects of genetic rescue were also observed for NGR, with significant MPH observed for nine (36%) of the genetic rescue crosses in both F_1_ and F_4_ (Table S4). Significant MPH following the genetic rescue was evident for both traits when averaged across recipient populations (Figure 3 and Table S6).

## 4 DISCUSSION

Theory predicts that while inbreeding can depress population fitness and increase the risk of extinction, the introduction of immigrants from other populations can genetically rescue small, inbred at‐risk populations by alleviating inbreeding depression and partly restore fitness (Hoffmann et al., 2021a, 2021b; Ingvarsson, 2001; Tallmon et al., 2004; Weeks et al., 2011, 2017). While the forced inbreeding induced in the current study's five recipient populations led to a decline in fitness, subsequent outcrossing with immigrants resulted in significant increases in both EAV and NGR (Figure 2). This is in agreement with numerous experimental studies showing that immigration can rescue small, inbred populations by rapidly improving fitness (Ball et al., 2000; Bijlsma et al., 2010; Bryant et al., 1999; Heber et al., 2012; Holleley et al., 2011; Hufbauer et al., 2015; Jensen et al., 2018; Spielman & Frankham, 1992; Swindell & Bouzat, 2006; Waite et al., 2005). Theoretical predictions state, that genetically divergent populations will produce offspring with fitness values intermediate between the two source populations. However, hybrid offspring may even experience heterosis if fitness exceeds mid‐parent values (Dlugosch et al., 2015). In the current study, in addition to an increase in fitness following immigration, hybrid offspring significantly outperformed mid‐parent fitness in F_1_ and F_4_ for NGR and in F_4_ for EAV (Figure 3), thus indicating the presence of heterosis following genetic rescue. This observation of heterosis following outcrossing with immigrants provides an indication of the genetic load present in the recipient populations prior to outcrossing, which is supported by the high level of inbreeding depression measured in these populations.

While most laboratory studies have examined the impacts of inbreeding and genetic rescue on traits closely related to fitness, e.g. survival or reproduction, the impact of inbreeding on behavioural traits has rarely been tested (but see Manenti et al., 2015; Miller et al., 1993), and we are not aware of any experimental studies examining the effects of genetic rescue on behavioural traits. Negative geotaxis, which we investigate here, has frequently been used to assess age‐related declines in locomotor activity (Grotewiel et al., 2005), and although NGR has been shown to be sensitive to the genetic background (Gargano et al., 2005), this trait has not before been investigated in relation to inbreeding nor genetic rescue. In the current study, outcrossing resulted in significant heterosis evident for both traits, albeit higher in EAV compared with NGR, especially in F_4_ (Figure 3). Nonetheless, while inbreeding depression and the beneficial effects of genetic rescue observed in the current study were more pronounced when examined using EAV, immigration did result in significant heterosis in the behavioural trait (i.e. NGR) in more than one‐third of the crosses in the F_1_ and F_4_ generation (Table S4). The observation of inbreeding depression and subsequent heterosis (following genetic rescue) in NGR might be of importance from an ecological point of view, since the ability to behaviourally respond to a stimulus is crucial, e.g. for predator avoidance and success in capturing prey in many species (Reed, 1999). Additionally, since inbreeding appears to impair the negative geotaxis behaviour and that such performance declines with age (Gargano et al., 2005), inbreeding may make individuals appear physiological older and thus perform worse than outbred individuals—an effect that we show to be partly reversible through outcrossing. The ability to measure the impacts of genetic rescue across both fitness and behavioural traits has potentially great implications for future conservation management, as the relative increase in fitness following outcrossing is not universal for all components of fitness (Whiteley et al., 2015). By including measurements on both a reproduction trait and a behavioural trait, the beneficial effects of immigration may be more easily detected and may provide a more comprehensive estimate of total fitness effects.

The five recipient populations showed different levels of heterosis, supported by the highly significant effect of the recipient population on the rescue effect (Table 1). This suggests that the rescue effect is greatly dependent on the population being rescued, which is consistent with findings in other studies showing that the effect of genetic rescue displays a strong population dependency (Bijlsma et al., 2010; Escobar et al., 2008; Jensen et al., 2018; Pickup et al., 2013). In addition, we found that the transgenerational effects depended on the recipient population as evident by strong interaction in the mixed models. Thus, population‐specific effects of genetic rescue remain a challenge for conservation geneticists, and the success of translocation efforts depends in part on the ability to predict these population‐specific effects.

As most experimental studies on genetic rescue have assessed its effects on the F_1_ offspring, studies extending beyond the first generation are rare (for a review see Edmands, 2007). In the current study, the effects of immigration were persistent with a beneficial effect of genetic rescue for both traits in both the F_1_ and F_4_ generation (Figures 2 and 3). Additionally, for EAV, mean fitness and MPH were observed to be, respectively, 21% and 287% higher in F_4_ when compared to F_1_. While the beneficial effects of genetic rescue have been shown to persist across multiple generations (Bijlsma et al., 2010; Frankham, 2016; Frankham et al., 2017), other studies have shown heterosis to decline following F_1_ (Heber et al., 2012; Jensen et al., 2018). Based on theoretical predictions, heterosis is expected to peak in F_1_ (due to maximum heterozygosity), followed by a decline in later generations due to re‐accumulation of genetic load and possible expression of outbreeding depression (Dlugosch et al., 2015; Edmands, 2007; Tallmon et al., 2004; Waller, 2015; Whiteley et al., 2015). However, our observation of a persistent and increasingly beneficial effect of genetic rescue on fitness from F_1_ to F_4_ (for EAV) may be caused by evolutionary adaptation, enabled by the introduction of new genetic variation, thus leading to a greater ability to respond to selection (Falconer & Mackay, 1996; Ørsted et al., 2019).

We found strong evidence for inbreeding depression prior to genetic rescue, as the large majority of the inbred lines showed evidence for inbreeding depression in EAV or NGR (Figure S3). Additionally, the inbred lines displayed inbreeding depression rates (B) for EAV in line with similar studies on *D*. *melanogaster* (Bundgaard et al., 2021; Enders & Nunney, 2010, 2012; Schou et al., 2015). Nonetheless, a large variation in inbreeding depression estimates was seen among different inbred lines, for both traits, despite an expected constant inbreeding level. While some inbred lines showed severe inbreeding depression (δ >> 0), others performed well and a few even outperformed the outbred control lines (δ < 0) (Figure S3). This is in line with several other studies showing large phenotypic variation across replicate lines with the same expected level of inbreeding (Fowler & Whitlock, 1999; Kristensen et al., 2003; Mikkelsen et al., 2010; Ørsted et al., 2019, 2022; Reed et al., 2002; Whitlock & Fowler, 1996; Wright et al., 2008). This illustrates that stochastic processes, such as the experimental bottlenecks performed in this study, can have population‐specific (or line‐specific) outcomes in terms of the severity of inbreeding depression (Bouzat, 2010; Ørsted et al., 2019).

When measuring inbreeding depression across multiple traits, it is common for some traits to show higher levels of inbreeding depression than others. For instance, traits closely related to fitness generally exhibit higher levels of inbreeding depression than morphological traits, likely due to the larger proportion of directional dominance in such traits (DeRose & Roff, 1999; Kristensen & Sørensen, 2005; Mikkelsen et al., 2010; Schou et al., 2015; Wright et al., 2008). In the current study, median inbreeding depression was approximately 2–19 times higher for EAV than for NGR (Table S3), the inbreeding depression rate was approximately three times higher for EAV compared with NGR, and fewer lines exhibited inbreeding depression in NGR compared with EAV (Figure S3). Furthermore, the estimated mean inbreeding depression rate for NGR (B = 0.282) is generally below that of several *Drosophila* fitness‐related traits seen in the literature (for a review see Armbruster & Reed, 2005). While EAV is seen as a trait closely linked to fitness, it can be argued that NGR is a behavioural trait more distantly related to fitness and the trait therefore is expected to harbour less directional dominance. This may explain the lesser inbreeding depression observed in NGR compared with EAV.

We observed a positive, although relatively weak, correlation between measures of inbreeding depression for EAV and NGR. Thus, while inbreeding may cause inbreeding depression in multiple traits simultaneously, inbreeding depression in one trait may have little value in predicting inbreeding depression in another trait (Kristensen et al., 2011), which is important to keep in mind when monitoring and managing endangered populations. Additionally, this underlines the importance of evaluating the impacts of inbreeding across multiple traits, as these can be combined to provide a more sensitive and ecologically relevant indicator of inbreeding depression (Keller & Waller, 2002).

## 5 CONCLUSION

The overall objective of this study was to examine the impacts of inbreeding and genetic rescue on egg‐to‐adult viability (EAV) and negative geotaxis response (NGR) using replicated crosses between inbred lines of *D*. *melanogaster*. Based on the results we conclude (1) inbreeding led to a significant reduction in fitness with the majority of inbred lines showing evidence for inbreeding depression in both EAV and NGR, (2) median inbreeding depression was up to 19 times higher for EAV compared with NGR. Furthermore, inbreeding depression in EAV displayed a positive, although relatively weak, correlation with inbreeding depression in NGR, suggesting that inbreeding depression in EAV has limited power for predicting inbreeding effects in behavioural traits and (3) while inbreeding led to a decline in fitness, subsequent outcrossing resulted in significantly increased performance in both EAV and NGR. Additionally, the beneficial effects of genetic rescue persisted across multiple generations and even showed an average increase from F_1_ to F_4_ in egg‐to‐adult viability. The observation of heterosis in a behavioural trait following genetic rescue is to our knowledge a novel finding, which may provide crucial insight into which traits to investigate when monitoring the effects of inbreeding and genetic rescue. In conclusion, these results strongly support the potential use of genetic rescue as an important future tool in managing threatened populations.

## CONFLICTS OF INTEREST

The authors have no conflicts of interest to declare that are relevant to the content of this article.

## AUTHOR CONTRIBUTIONS

All authors contributed to the study conception and design. Data collection was performed by DBJ. Analysis was performed by DBJ and MØ. DBJ wrote the first draft, and all authors commented on previous versions of the manuscript. All authors approved the final manuscript.

## Supporting information

Supplementary Material

## Data Availability

Supplementary material is available online. Data is openly available in the Dryad Digital Repository at: https://doi.org/10.5061/dryad.0cfxpnw50
