# Supplementary material for: Sustained positive consequences of genetic rescue of fitness and behavioural traits in inbred populations of Drosophila melanogaster
Source: J Evol Biol. 2022 May 9;35(6):868–78. doi: 10.1111/jeb.14015 (PMC9325394; doi:10.1111/jeb.14015)
Supplement: Supplementary file 1 — Supplementary Material [file JEB-35-868-s001.docx]

**Supporting information for:**

**Sustained positive consequences of genetic rescue of fitness and behavioural traits in inbred populations of *Drosophila melanogaster***

**Contents:**

**Fig. S1** Egg-to-adult viability (EAV) for the five recipient, five donor, and five outbred control lines prior to the genetic rescue experiment.

**Fig. S2** The rapid iterative negative geotaxis (RING) apparatus.

**Fig. S3** Distribution of the mean inbreeding depression (δ) in (a) egg-to-adult viability (EAV) for 110 inbred lines and (b) negative geotaxis response (NGR) for 106 inbred lines.

**Table S1** Correction for the effect of RING assay trial number on NGR.

**Table S2** Spearman correlation between inbreeding depression in egg-to-adult viability (EAV) and negative geotaxis response (NGR).

**Table S3** Median inbreeding depression in egg-to-adult viability (EAV) and negative geotaxis response (NGR) across all inbred lines.

**Table S4** Mid-parent heterosis (MPH) of each genetic rescue cross measured by egg-to-adult viability (EAV) and negative geotaxis response (NGR).

**Table S5** Mean fitness for egg-to-adult viability (EAV) and negative geotaxis response (NGR) across generations.

**Table S6** Mean mid-parent heterosis (MPH) for egg-to-adult viability (EAV) and negative geotaxis response (NGR) across generations.

**Table S7** Fitness values of recipient populations, measured by egg-to-adult viability (EAV) and negative geotaxis response (NGR), prior to genetic rescue (Pre-GR) and post genetic rescue (F_1_ and F_4_).


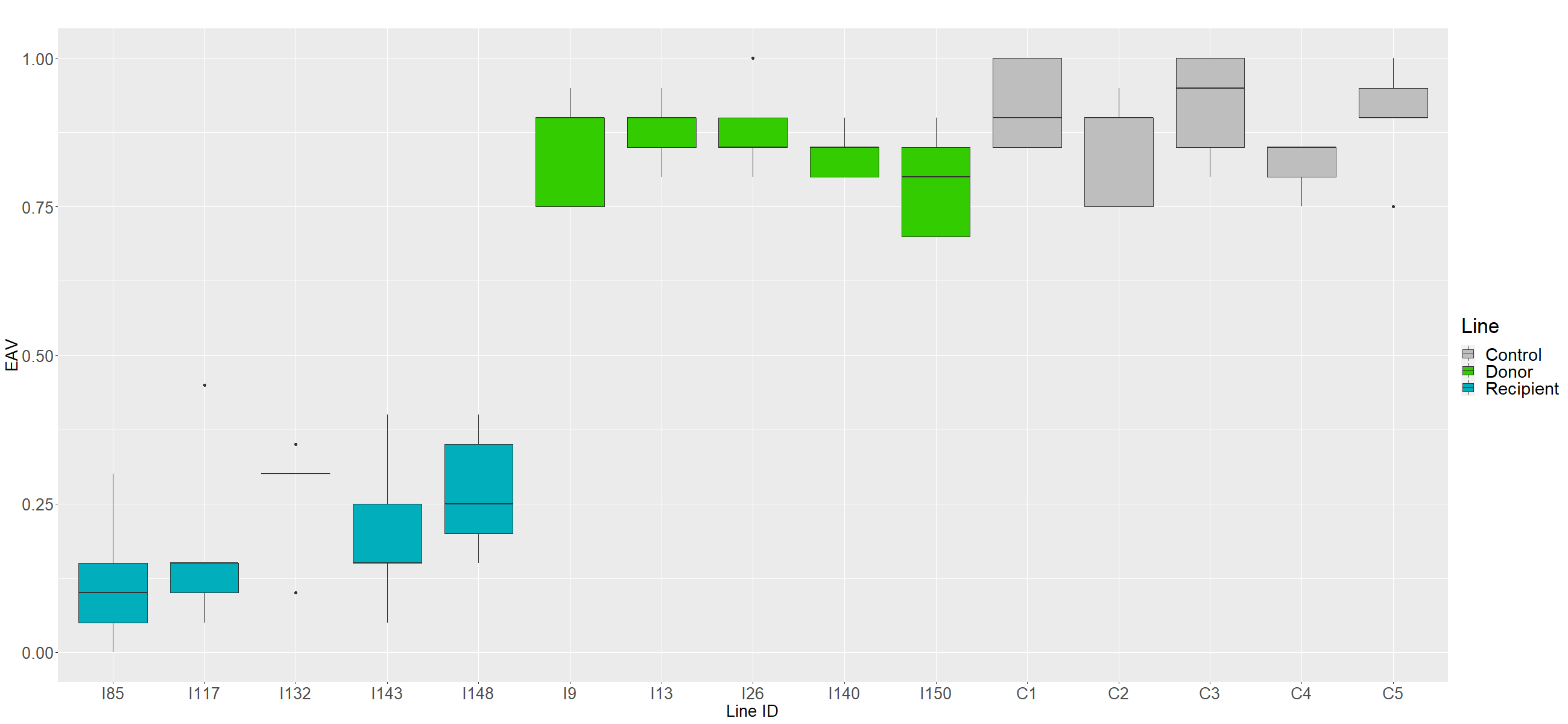


**Fig. S1.** Egg-to-adult viability (EAV) for the five recipient, five donor, and five outbred control lines prior to the genetic rescue experiment. Colours highlight the selected lines used in the genetic rescue experiment as recipient (blue; I85, I117, I132, I143 and I148) and donor (green; I9, I13, I26, I140 and I150) populations. Additionally, control populations (gray) are also highlighted.


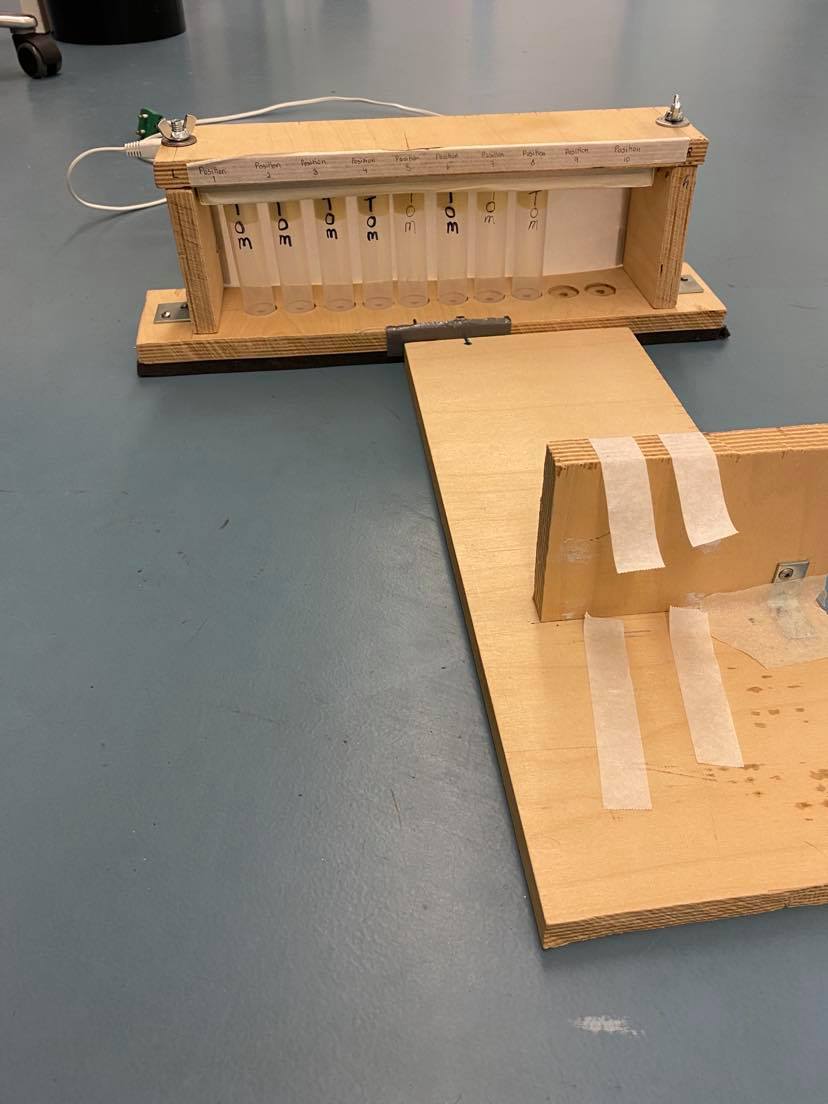

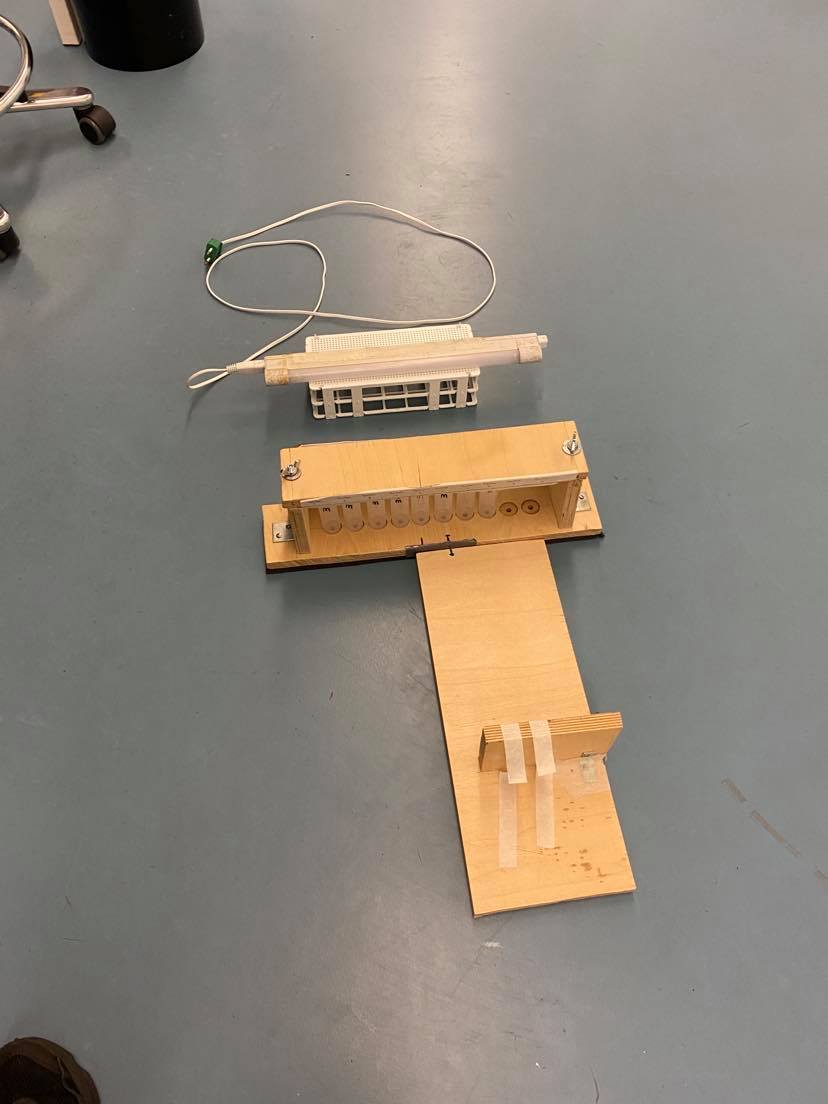

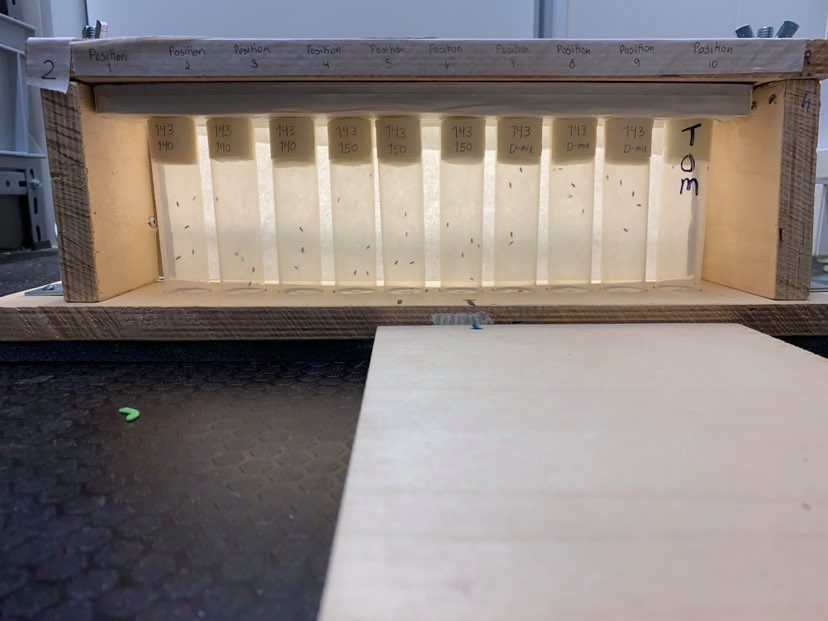


**Fig. S2.** The rapid iterative negative geotaxis (RING) apparatus. The RING apparatus consists of a wooden box with an open face and a removable lid held in place with hinges on each side, fastened using a bolt and a nut. The floor of the apparatus supports ten 46.63 mL vials (25 mm x 95 mm) (VWR™, PA) with a flat bottom, used as negative geotaxis tubes. The apparatus lid is lined with foam to ensure that each negative geotaxis tube is fastened in the apparatus. Adjacent to the face of the apparatus is a wooden board with a vertical board attached, allowing placement of a camera apparatus 30 cm in front of the RING apparatus. The camera apparatus was fastened to the wooded board using masking tape. To provide uniform lightning of the RING apparatus, a light source was placed behind a white opaque filter attached to the backside of the apparatus.

**a**


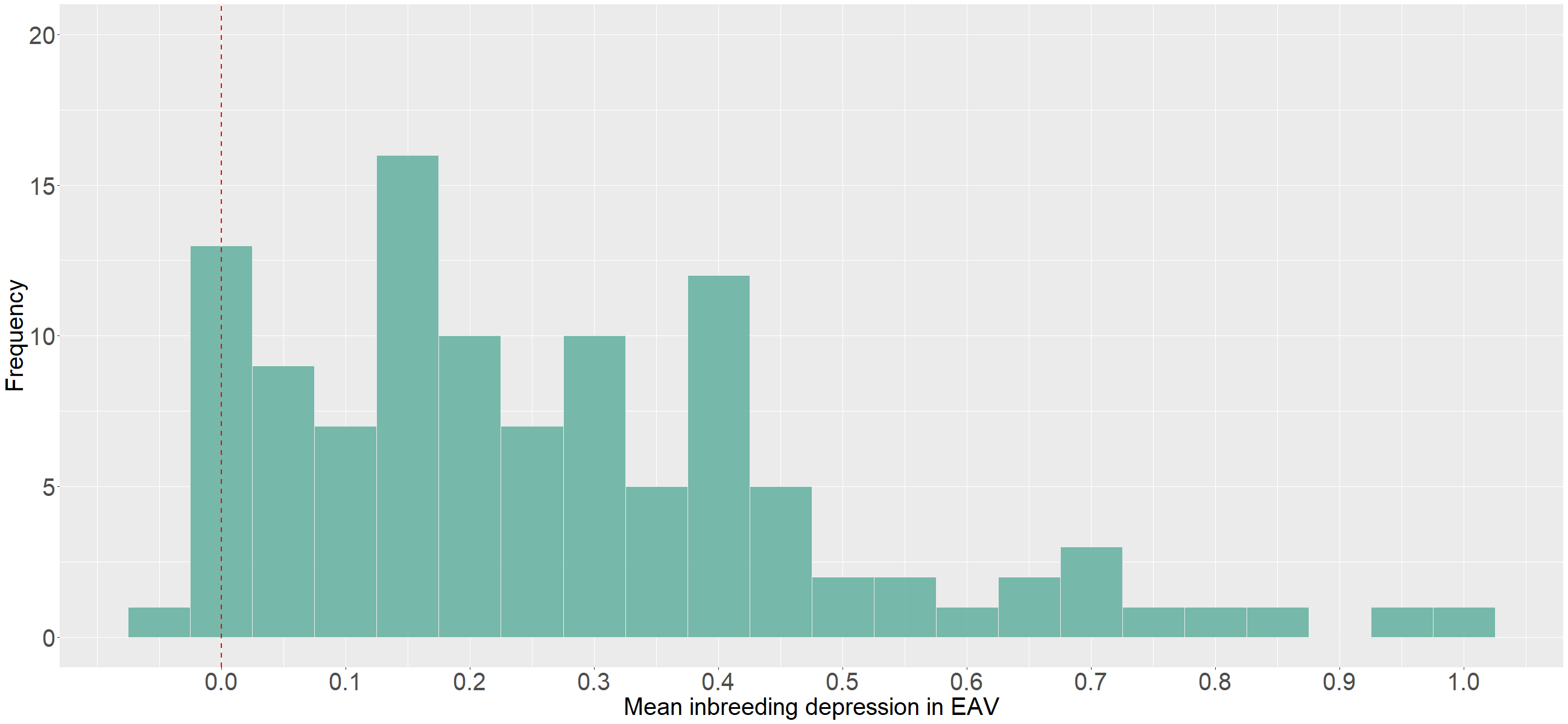


**b**


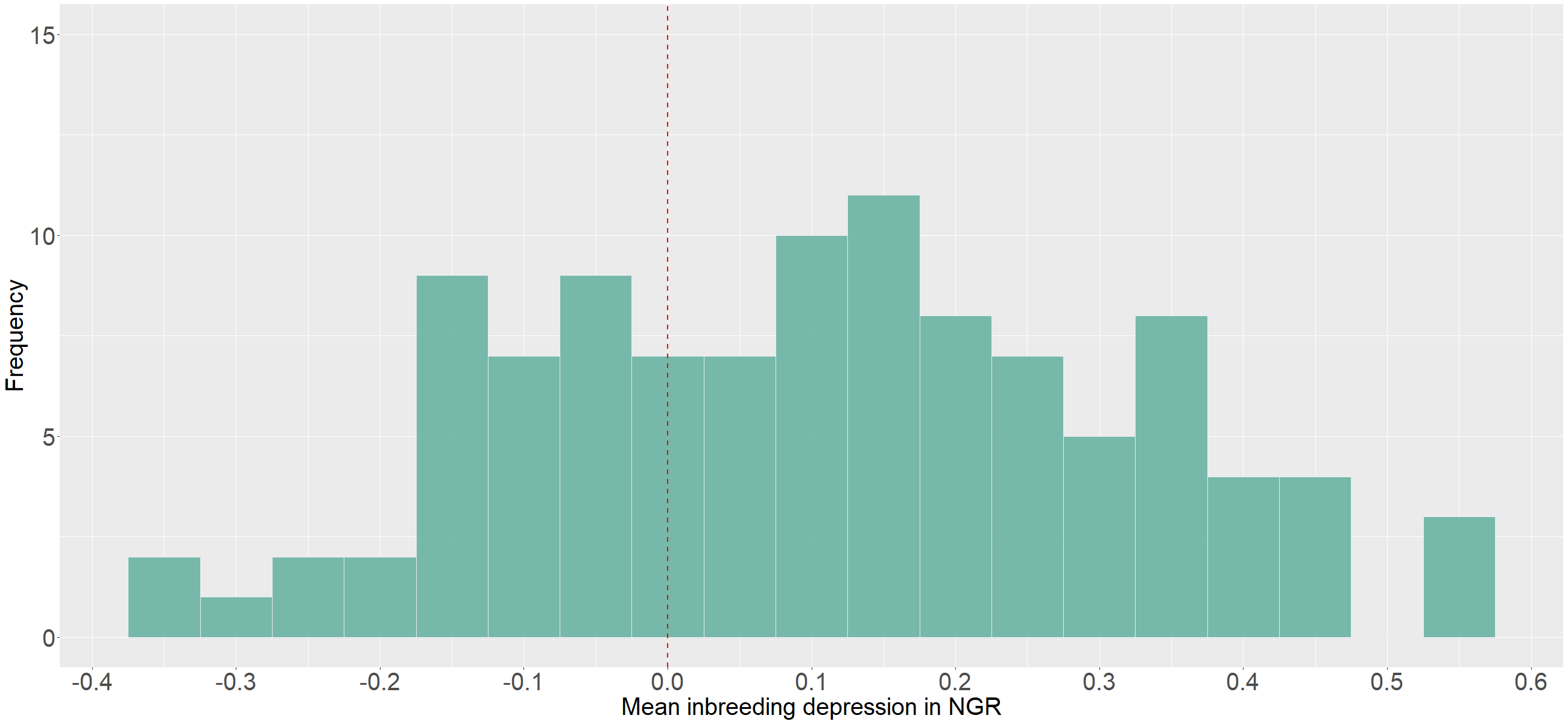


**Fig. S3.** Distribution of the mean inbreeding depression (δ) in (**a**) egg-to-adult viability (EAV) for 110 inbred lines and (**b**) negative geotaxis response (NGR) for 106 inbred lines. Values to the right of the vertical red dotted line express some degree of inbreeding depression.

| Generation | Line | p-value | Slope |
| --- | --- | --- | --- |
| Pre-GR | I20 | 0.044^*^ | 0.372 |
|  | I22 | 0.039^*^ | -0.226 |
|  | I77 | 0.033^*^ | 0.495 |
|  | I82 | 0.030^*^ | 0.309 |
|  | I95 | 0.021^*^ | -0.569 |
| Post-GR (F_1_) | I132 x I9 | 0.040^*^ | -0.476 |
|  | I132 x I132 | 0.003^**^ | -0.222 |
|  | I148 x I13 | 0.036^*^ | -0.269 |
| Post-GR (F_4_) | I117 x I150 | 0.050^*^ | -0.168 |
|  | I150 x I150 | 0.014^*^ | -0.496 |

**Table S1.** Correction for the effect of RING assay trial number on negative geotaxis response (NGR). To take into account the possibility of the flies showing fatigue or disorientation during consecutive RING assay trials (i.e. showing a reduced NGR as the number of trials increased), all lines were assessed for a possible correlation between NGR and trial number. This was done by calculating the median NGR for each of the five trails (without regard to the number of replicate vials), for each line, and plotting these as a function of trial number. Lines showing a significant linear relationship with the trial number were corrected by estimating the effect of trial number with a linear model and adjusting the NGR accordingly. Prior to the genetic rescue experiment (Pre-GR), 4.5% (5/111) of the lines showed a significant correlation with trial number, whilst 7.5% (3/40) and 5% (2/40) of the genetic rescue crosses showed a correlation with trial number in the F_1_ and F_4_ generation after the genetic rescue experiment was initiated (Post-GR), respectively. Significance levels are denoted by asterisks: *** p < 0.001; ** p < 0.01; * p < 0.05. A correction for the effect of RING assay trial number on NGR seen for these lines was carried out by multiplying the slope of the correlation with the trial number minus 1, and adding or subtracting this value to the measured NGR values for the specific line (depending on if the correlation was negative or positive, respectively). As an example, the slope for the correlation between NGR and RING assay trial number for line I22 was -0.226. For NGR values measured in trial number two, 0.266 multiplied by one was added to all NGR values, and 0.266 multiplied by two was added to NGR values measured in trial number three and so on. Additionally, after adjusting the NGR values for an effect of trial number, adjusted values below 0 were given the value 0, while values above the height of the vials used in the RING assay were adjusted to the maximum height of said vial.

**Table S2.** Spearman correlation between inbreeding depression in egg-to-adult viability (EAV) and negative geotaxis response (NGR). Results of the correlation between inbreeding depression in the two investigated traits prior to the genetic rescue experiment (Pre-GR) and in F_1_ and F_4_ generation after initiation of the genetic rescue experiment (Post-GR). For each analysis, sample size (n), Spearman's rank correlation coefficient (r_s_) , and p-value are shown. Significance levels are denoted by asterisks: *** p < 0.001; ** p < 0.01; * p < 0.05.

| Generation | Correlation between inbreeding depression in EAV and NGR | | |
| --- | --- | --- | --- |
|  | n (number of lines) | r_s_ | p |
| Pre-GR | 106 | 0.263 | 0.006** |
| Post-GR (F_1_) | 35 | 0.296 | 0.084 |
| Post-GR (F_4_) | 35 | 0.369 | 0.030* |

**Table S3.** Median inbreeding depression in egg-to-adult viability (EAV) and negative geotaxis response (NGR) across all inbred lines. Sample size (n), median, and variance for inbreeding depression in EAV and NGR prior to the genetic rescue experiment (Pre-GR) and in F_1_ and F_4_ generation after initiation of the genetic rescue experiment (Post-GR). Results of a Mann-Whitney U test testing for a difference between the two fitness traits are also shown. Significance levels are denoted by asterisks: *** p < 0.001; ** p < 0.01; * p < 0.05.

| Generation | Fitness assay | n (number of lines) | Median | Variance | Mann-Whitney U test |
| --- | --- | --- | --- | --- | --- |
| Pre-GR | EAV | 106 | 0.218 | 0.042 | W = 8360 p < 0.001^***^ |
|  | NGR | 106 | 0.107 | 0.043 |  |
| Post-GR (F_1_) | EAV | 35 | 0.248 | 0.080 | W = 870 p = 0.003^**^ |
|  | NGR | 35 | 0.065 | 0.031 |  |
| Post-GR (F_4_) | EAV | 35 | 0.097 | 0.055 | W = 930 p < 0.001^***^ |
|  | NGR | 35 | -0.005 | 0.017 |  |

**Table S4.** Mid-parent heterosis (MPH) of each genetic rescue cross measured by egg-to-adult viability (EAV) and negative geotaxis response (NGR). Sample size (n), mean, and standard error (SE) of the rescue effect of each genetic rescue cross in the F_1_ and F_4_ generation (Gen.) after initiation of the genetic rescue experiment are shown. Results of a one-tailed one-sample Wilcoxon signed rank test, testing for offspring fitness being greater than mid-parent fitness, are also shown. Significance levels are denoted by asterisks: *** p < 0.001; ** p < 0.01; * p < 0.05.

|  |  |  | EAV | | | | NGR | | | |
| --- | --- | --- | --- | --- | --- | --- | --- | --- | --- | --- |
| Rec.pop. | Don.pop. | Gen. | n | Mean MPH | SE | p | n | Mean MPH | SE | p |
| I85 | I9 | F1 | 10 | 26.786 | 7.143 | 0.008^**^ | 5 | -12.034 | 6.480 | 0.938 |
|  |  | F4 | 10 | 55.455 | 4.381 | 0.003^**^ | 6 | 1.404 | 11.260 | 0.422 |
|  | I13 | F1 | 10 | 39.815 | 6.540 | 0.003^**^ | 6 | 21.304 | 3.448 | 0.016^*^ |
|  |  | F4 | 10 | 26.891 | 9.740 | 0.032^*^ | 6 | 24.758 | 5.793 | 0.016^*^ |
|  | I26 | F1 | 10 | 23.721 | 6.362 | 0.007^**^ | 6 | 32.483 | 4.255 | 0.016^*^ |
|  |  | F4 | 10 | 48.837 | 7.844 | 0.004^**^ | 6 | 33.994 | 9.227 | 0.031^*^ |
|  | I140 | F1 | 10 | -4.072 | 6.356 | 0.730 | 6 | -0.115 | 10.634 | 0.656 |
|  |  | F4 | 10 | 19.266 | 15.831 | 0.154 | 6 | 16.781 | 6.143 | 0.031^*^ |
|  | I150 | F1 | 10 | 17.117 | 8.387 | 0.075 | 6 | -0.445 | 8.713 | 0.422 |
|  |  | F4 | 10 | 60.550 | 4.159 | 0.003^**^ | 6 | 1.524 | 8.283 | 0.500 |
| I117 | I9 | F1 | 10 | -28.947 | 6.725 | 0.993 | 6 | 5.759 | 8.908 | 0.281 |
|  |  | F4 | 10 | 57.073 | 6.891 | 0.003^**^ | 6 | 1.223 | 4.794 | 0.422 |
|  | I13 | F1 | 10 | -39.091 | 4.505 | 0.998 | 5 | 25.823 | 6.993 | 0.031^*^ |
|  |  | F4 | 10 | 3.139 | 8.376 | 0.380 | 6 | 1.901 | 7.896 | 0.422 |
|  | I26 | F1 | 10 | -36.986 | 4.804 | 0.998 | 6 | -8.950 | 7.972 | 0.844 |
|  |  | F4 | 10 | 32.000 | 11.719 | 0.018^*^ | 6 | -5.453 | 8.591 | 0.719 |
|  | I140 | F1 | 10 | -17.333 | 7.018 | 0.980 | 6 | -18.967 | 6.346 | 0.999 |
|  |  | F4 | 10 | 51.724 | 13.070 | 0.007^**^ | 6 | 21.534 | 10.010 | 0.078 |
|  | I150 | F1 | 10 | -28.319 | 7.630 | 0.998 | 6 | 16.878 | 4.818 | 0.016^*^ |
|  |  | F4 | 10 | 29.064 | 11.132 | 0.021^*^ | 6 | 21.884 | 3.636 | 0.016^*^ |
| I132 | I9 | F1 | 10 | 53.587 | 2.756 | 0.003^**^ | 6 | 25.716 | 4.672 | 0.016^*^ |
|  |  | F4 | 10 | 39.286 | 9.988 | 0.009^**^ | 6 | -7.092 | 5.418 | 0.891 |
|  | I13 | F1 | 10 | 58.952 | 5.493 | 0.003^**^ | 6 | -7.941 | 8.079 | 0.844 |
|  |  | F4 | 10 | 32.231 | 8.798 | 0.009^**^ | 6 | 1.015 | 10.319 | 0.500 |
|  | I26 | F1 | 10 | 63.158 | 3.256 | 0.003^**^ | 6 | -27.194 | 9.457 | 0.969 |
|  |  | F4 | 10 | 51.598 | 6.834 | 0.003^**^ | 6 | 16.609 | 10.954 | 0.156 |
|  | I140 | F1 | 10 | 56.410 | 5.705 | 0.003^**^ | 6 | 5.730 | 7.036 | 0.219 |
|  |  | F4 | 10 | 64.865 | 4.464 | 0.003^**^ | 6 | 15.548 | 5.407 | 0.016^*^ |
|  | I150 | F1 | 10 | 47.234 | 10.769 | 0.005^**^ | 6 | 1.940 | 9.376 | 0.281 |
|  |  | F4 | 10 | 53.153 | 5.025 | 0.003^**^ | 6 | -10.542 | 6.967 | 0.922 |
| I143 | I9 | F1 | 9 | 28.689 | 10.936 | 0.016^*^ | 6 | 33.779 | 7.277 | 0.016^*^ |
|  |  | F4 | 10 | 52.294 | 13.344 | 0.008^**^ | 6 | 8.818 | 4.254 | 0.078 |
|  | I13 | F1 | 10 | 44.666 | 8.370 | 0.004^**^ | 6 | 35.435 | 6.500 | 0.016^*^ |
|  |  | F4 | 10 | 42.373 | 5.762 | 0.003^**^ | 6 | 25.914 | 4.437 | 0.016^*^ |
|  | I26 | F1 | 10 | 30.481 | 12.042 | 0.026^*^ | 6 | 33.743 | 5.709 | 0.016^*^ |
|  |  | F4 | 10 | 73.709 | 4.020 | 0.003^**^ | 6 | 30.969 | 6.011 | 0.016^*^ |
|  | I140 | F1 | 10 | 50.432 | 6.655 | 0.003^**^ | 6 | -12.670 | 9.029 | 0.844 |
|  |  | F4 | 10 | 42.593 | 7.459 | 0.003^**^ | 6 | 15.550 | 7.552 | 0.047^*^ |
|  | I150 | F1 | 10 | 44.132 | 7.876 | 0.004^**^ | 6 | 14.076 | 7.214 | 0.047^*^ |
|  |  | F4 | 10 | 59.259 | 10.124 | 0.004^**^ | 6 | -2.665 | 11.403 | 0.578 |
| I148 | I9 | F1 | 10 | -30.472 | 5.190 | 0.998 | 6 | -17.145 | 8.884 | 0.953 |
|  |  | F4 | 10 | 14.433 | 17.282 | 0.179 | 6 | 8.545 | 6.034 | 0.156 |
|  | I13 | F1 | 10 | -33.516 | 7.904 | 0.996 | 5 | 24.034 | 7.301 | 0.063 |
|  |  | F4 | 10 | 42.453 | 9.270 | 0.003^**^ | 6 | 16.906 | 4.027 | 0.016^*^ |
|  | I26 | F1 | 10 | -19.643 | 6.099 | 0.998 | 6 | 12.202 | 6.585 | 0.078 |
|  |  | F4 | 10 | 15.344 | 10.866 | 0.077 | 6 | 2.184 | 8.955 | 0.500 |
|  | I140 | F1 | 10 | -40.870 | 5.153 | 0.998 | 5 | -1.416 | 5.115 | 0.688 |
|  |  | F4 | 10 | 40.625 | 9.852 | 0.007^**^ | 6 | 3.606 | 11.283 | 0.422 |
|  | I150 | F1 | 10 | -37.662 | 8.039 | 0.997 | 5 | 16.103 | 9.139 | 0.094 |
|  |  | F4 | 10 | 31.250 | 3.541 | 0.003^**^ | 6 | -1.534 | 10.867 | 0.422 |

**Table S5** Mean fitness for egg-to-adult viability (EAV) and negative geotaxis response (NGR) across generations. Values are averaged across all recipient populations in the generation prior to genetic rescue (Pre-GR), and averaged across all genetic rescue crosses in the F_1_ and F_4_ generation. Values for each recipient population are shown in Table S7.

|  | EAV | | | | NGR | |
| --- | --- | --- | --- | --- | --- | --- |
| Gen. | n | Mean | SE | n | Mean | SE |
| Pre-GR | 25 | 0.208 | 0.025 | 6 | 2.955 | 0.183 |
| F1 | 249 | 0.626 | 0.093 | 145 | 3.707 | 0.093 |
| F4 | 250 | 0.758 | 0.037 | 150 | 3.629 | 0.104 |

**Table S6** Mean mid-parent heterosis (MPH) for egg-to-adult viability (EAV) and negative geotaxis response (NGR) across generations. Values are averaged across all recipient populations in the two generations following genetic rescue (F_1_ and F_4_). Results of one-tailed one-sample Wilcoxon signed rank tests, testing whether offspring fitness is greater than mid-parent fitness (MPH > 0), are also shown. Additionally, results of two-sample paired Wilcoxon signed rank tests, testing for a difference between heterosis in generations (F_1_ and F_4_), are also shown. Significance levels are denoted by asterisks: *** p < 0.001; ** p < 0.01; * p < 0.05.

| Trait | Generation | n | Mean MPH (%) | SE | *p* (MPH > 0) | *p* (Generation) |
| --- | --- | --- | --- | --- | --- | --- |
| EAV | F1 | 25 | 10.731 | 6.869 | 0.063 | <0.001*** |
|  | F4 | 25 | 41.579 | 8.791 | <0.001^***^ |  |
| NGR | F1 | 25 | 7.921 | 7.198 | 0.031^*^ | 0.792 |
|  | F4 | 25 | 9.735 | 7.581 | <0.001^***^ |  |

**Table S7**. Fitness values of recipient populations, measured by egg-to-adult viability (EAV) and negative geotaxis response (NGR), prior to genetic rescue (Pre-GR) and post genetic rescue (F_1_ and F_4_). Sample size (n), mean, and standard error (SE) of fitness values are shown.

|  |  |  | EAV | | | | NGR | |
| --- | --- | --- | --- | --- | --- | --- | --- | --- |
| Rec.pop. | Don.pop. | Gen. | n | Mean | SE | n | Mean | SE |
| I85 | I85 | Pre-GR | 5 | 0.120 | 0.051 | 1 | 2.567 | *NA* |
|  | I9 | F1 | 10 | 0.710 | 0.040 | 5 | 3.189 | 0.235 |
|  |  | F4 | 10 | 0.885 | 0.024 | 6 | 3.533 | 0.392 |
|  | I13 | F1 | 10 | 0.755 | 0.035 | 6 | 4.293 | 0.122 |
|  |  | F4 | 10 | 0.755 | 0.058 | 6 | 4.422 | 0.205 |
|  | I26 | F1 | 10 | 0.665 | 0.034 | 6 | 4.061 | 0.130 |
|  |  | F4 | 10 | 0.800 | 0.042 | 6 | 4.104 | 0.283 |
|  | I140 | F1 | 10 | 0.530 | 0.035 | 6 | 3.278 | 0.349 |
|  |  | F4 | 10 | 0.650 | 0.086 | 6 | 3.874 | 0.204 |
|  | I150 | F1 | 10 | 0.650 | 0.047 | 6 | 3.172 | 0.278 |
|  |  | F4 | 10 | 0.875 | 0.023 | 6 | 3.692 | 0.301 |
| I117 | I117 | Pre-GR | 5 | 0.180 | 0.070 | 1 | 2.936 | *NA* |
|  | I9 | F1 | 10 | 0.405 | 0.038 | 6 | 3.833 | 0.323 |
|  |  | F4 | 10 | 0.805 | 0.035 | 6 | 3.358 | 0.159 |
|  | I13 | F1 | 10 | 0.335 | 0.025 | 5 | 4.450 | 0.247 |
|  |  | F4 | 10 | 0.575 | 0.047 | 6 | 3.442 | 0.267 |
|  | I26 | F1 | 10 | 0.345 | 0.026 | 6 | 2.789 | 0.244 |
|  |  | F4 | 10 | 0.660 | 0.059 | 6 | 2.738 | 0.248 |
|  | I140 | F1 | 10 | 0.465 | 0.039 | 6 | 2.657 | 0.208 |
|  |  | F4 | 10 | 0.770 | 0.066 | 6 | 3.829 | 0.315 |
|  | I150 | F1 | 10 | 0.405 | 0.043 | 6 | 3.721 | 0.153 |
|  |  | F4 | 10 | 0.665 | 0.056 | 6 | 4.229 | 0.126 |
| I132 | I132 | Pre-GR | 5 | 0.270 | 0.044 | 2 | 3.181 | 0.603 |
|  | I9 | F1 | 10 | 0.910 | 0.016 | 6 | 5.439 | 0.202 |
|  |  | F4 | 10 | 0.780 | 0.056 | 6 | 3.260 | 0.190 |
|  | I13 | F1 | 10 | 0.910 | 0.031 | 6 | 3.902 | 0.342 |
|  |  | F4 | 10 | 0.800 | 0.053 | 6 | 3.605 | 0.368 |
|  | I26 | F1 | 10 | 0.930 | 0.019 | 6 | 2.742 | 0.356 |
|  |  | F4 | 10 | 0.830 | 0.037 | 6 | 3.600 | 0.338 |
|  | I140 | F1 | 10 | 0.915 | 0.033 | 6 | 4.210 | 0.280 |
|  |  | F4 | 10 | 0.915 | 0.025 | 6 | 3.862 | 0.181 |
|  | I150 | F1 | 10 | 0.865 | 0.063 | 6 | 3.962 | 0.364 |
|  |  | F4 | 10 | 0.850 | 0.028 | 6 | 3.275 | 0.255 |
| I143 | I143 | Pre-GR | 5 | 0.200 | 0.059 | 1 | 2.327 | *NA* |
|  | I9 | F1 | 9 | 0.739 | 0.063 | 6 | 4.502 | 0.245 |
|  |  | F4 | 10 | 0.830 | 0.073 | 6 | 3.427 | 0.134 |
|  | I13 | F1 | 10 | 0.805 | 0.047 | 6 | 4.440 | 0.213 |
|  |  | F4 | 10 | 0.840 | 0.034 | 6 | 4.041 | 0.142 |
|  | I26 | F1 | 10 | 0.720 | 0.066 | 6 | 3.752 | 0.160 |
|  |  | F4 | 10 | 0.925 | 0.021 | 6 | 3.573 | 0.164 |
|  | I140 | F1 | 10 | 0.850 | 0.037 | 6 | 2.636 | 0.273 |
|  |  | F4 | 10 | 0.770 | 0.040 | 6 | 3.446 | 0.225 |
|  | I150 | F1 | 10 | 0.820 | 0.045 | 6 | 3.338 | 0.211 |
|  |  | F4 | 10 | 0.860 | 0.055 | 6 | 3.213 | 0.376 |
| I143 | I143 | Pre-GR | 5 | 0.270 | 0.046 | 1 | 3.536 | *NA* |
|  | I9 | F1 | 10 | 0.405 | 0.030 | 6 | 3.111 | 0.334 |
|  |  | F4 | 10 | 0.555 | 0.084 | 6 | 3.807 | 0.212 |
|  | I13 | F1 | 10 | 0.375 | 0.044 | 5 | 4.548 | 0.268 |
|  |  | F4 | 10 | 0.755 | 0.049 | 6 | 4.170 | 0.144 |
|  | I26 | F1 | 10 | 0.450 | 0.034 | 6 | 3.583 | 0.210 |
|  |  | F4 | 10 | 0.545 | 0.051 | 6 | 3.153 | 0.276 |
|  | I140 | F1 | 10 | 0.340 | 0.030 | 5 | 3.361 | 0.174 |
|  |  | F4 | 10 | 0.675 | 0.047 | 6 | 3.461 | 0.377 |
|  | I150 | F1 | 10 | 0.360 | 0.046 | 5 | 3.848 | 0.303 |
|  |  | F4 | 10 | 0.630 | 0.017 | 6 | 3.603 | 0.398 |
